# Strengthening of Al-Fe_3_Al composites by the generation of harmonic structures

**DOI:** 10.1038/s41598-018-24824-y

**Published:** 2018-04-24

**Authors:** R. N. Shahid, S. Scudino

**Affiliations:** Institute for Complex Materials, IFW Dresden, Helmholtzstraße 20, D-01069 Dresden, Germany

## Abstract

Strengthening of alloys can be efficiently attained by the creation of harmonic structures: bimodal microstructures generated by controlled milling of the particulate precursors, which consist of coarse-grained cores embedded in a continuous fine-grained matrix. Here, we extend the concept of harmonic structures to metal matrix composites and analyze the effectiveness of such bimodal microstructures for strengthening composites consisting of a pure Al matrix reinforced with Fe_3_Al particles. Preferential microstructural refinement limited to the surface of the particles, where the Fe_3_Al phase is progressively fragmented, occurs during ball milling of the Al-Fe_3_Al composite powder mixtures. The refined surface becomes the continuous fine-grained matrix that encloses macro-regions with coarser reinforcing particles in the harmonic composites synthesized during subsequent powder consolidation. The generation of the bimodal microstructure has a significant influence on the strength of the harmonic composites, which exceeds that of the conventional material by a factor of 2 while retaining considerable plastic deformation. Finally, modeling of the mechanical properties indicates that the strength of the harmonic composites can be accurately described by taking into account both the volume fraction of reinforcement and the characteristic microstructural features describing the harmonic structure.

## Introduction

Aluminum matrix composites (AMCs) are attractive materials for application in automotive, aircraft and transportation industries because of their low density, high specific strength, stiffness, workability, good thermal stability and enhanced resistance to wear, corrosion and fatigue^1–5^. Particulate-reinforced AMCs synthesized by solid-state powder metallurgy (e.g. through pressure-assisted sintering) are of particular interest thanks to the excellent control over fundamental microstructural features, including size, morphology and distribution of the reinforcing phase^6–8^, which allows the development of advanced materials with customized properties.

The strengthening of composites is based on the incorporation of a hard phase in a metallic matrix to improve the properties compared to the unreinforced material^9–12^. According to the shear lag model, strengthening of a composite depends on the load bearing ability of the reinforcement: the soft matrix transfers the applied stress to the reinforcing particles, which share the stress and strengthen the composite^13–15^. Simultaneously, the addition of the reinforcement generates microstructural variations in the matrix, which enhance the strength of the composites by Orowan strengthening, matrix partitioning and dislocation multiplication^16–19^. As a composite is cooled down during synthesis, the difference between the coefficient of thermal expansion of matrix and reinforcement leads to the formation of dislocations at the matrix-reinforcement interface. These thermally-induced dislocations as well improve the strength of the composites^20–24^.

Both load bearing and microstructural strengthening effects coexist and are related to the distribution, volume fraction and size of the reinforcement^22,25^. The strength increases with increasing the reinforcement volume fraction as well as by reducing the size of the reinforcing phase^5,19,26–32^, provided that the reinforcing particles are homogeneously dispersed in the matrix and no significant reinforcement clustering occurs. Particular important for large volume fractions of reinforcement and/or small particle size is the reduction of the matrix ligament size (λ), which can be regarded as the average distance between adjacent reinforcing particles, and results in a similar strengthening as the Hall-Petch effect observed for grain refinement^22^.

More unconventional strengthening methods include (*i*) controlled interfacial reactions between matrix and reinforcement during processing or, subsequently, by post processing heat treatments to obtain microstructural changes and phase transformations capable to further enhance the strength of the composites^11,33–40^ and (*ii*) microstructural modifications induced by ball milling of the composite powder mixtures^41,42^. Ball milling, which consists of high-energy collisions of the grinding balls with the particles, is a flexible and commonly used method of de-agglomeration, size reduction and homogeneous dispersion of the reinforcing particles within the matrix^43–48^. Protracted ball milling leads to microstructural refinement by deforming, fracturing and cold welding of the composite powder^41^. Such microstructural modifications can induce substantial strengthening in composites without the need to increase the volume fraction of the reinforcing phase^41,42^.

Materials strengthening while retaining appreciable plastic deformation can be achieved by generating harmonic structures: bimodal heterogeneous microstructures consisting of coarse-grained cores embedded in a continuous fine-grained matrix^49^. Such microstructures are synthesized by solid-state powder processing, for example by controlled milling of the particles followed by powder consolidation^49,50^. The harmonic structure and its gradual variation of size from coarse to fine leads to enhanced strength and toughness for a variety of materials, including pure Ti, Ti-6Al-4V, steel and Co-Cr-Mo^49–52^. The advantage of the harmonic structures over other bimodal arrangements resulting from simply mixing coarse- and fine-grained particles is the creation of controlled heterogeneous microstructures, which avoids the irregular distribution of the coarse- and fine-grained areas and leads to a good reproducibility of the mechanical properties^49^.

In this work, we aim to extend the concept of harmonic structures to metal matrix composites by analyzing the effectiveness of such bimodal microstructures as a strengthening method for composites consisting of a pure Al matrix reinforced with Fe_3_Al particles. Iron aluminides, and in particular Fe_3_Al, are prospective substitutes for ceramics as strengthening agents in AMCs because of lower cost and remarkable mechanical properties along with excellent resistance to corrosion under different aggressive environments^53–59^. Accordingly, the purpose of the present study is to examine the microstructural variations induced by ball milling of the Al-Fe_3_Al composite powder mixtures and how such variations influence the resulting microstructure and mechanical response of the bulk composite specimens synthesized by hot-pressing.

## Ball milling of Al-Fe_3_Al powder mixtures

**Figure 1:**
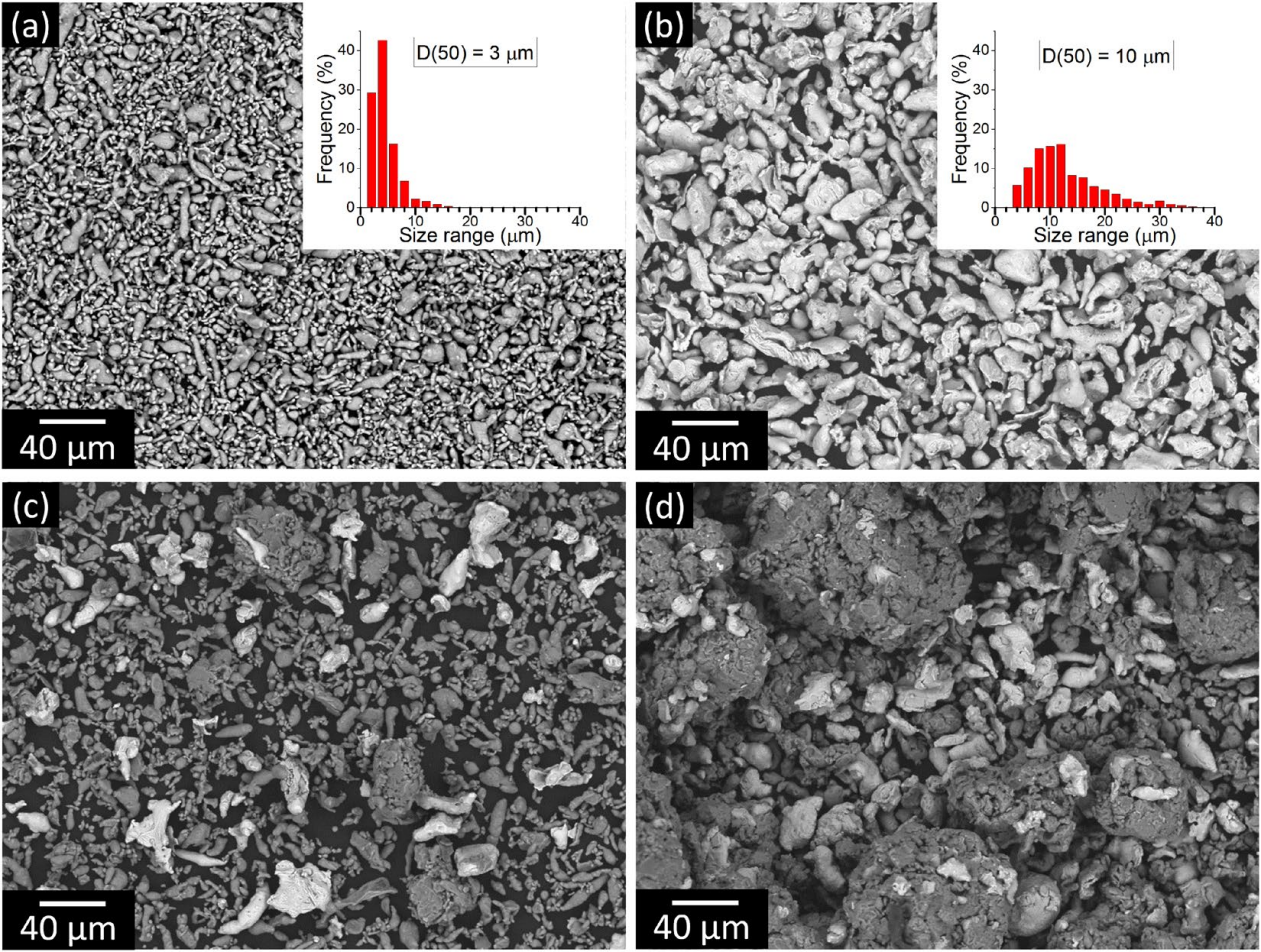
Initial particle morphology. SEM micrographs and corresponding particle size distribution of (**a**) pure aluminum and (**b**) Fe_3_Al. Particle morphology for the Al-Fe_3_Al powder mixtures milled for (**c**) 1 and (**d**) 5 h.

**Figure 2:**
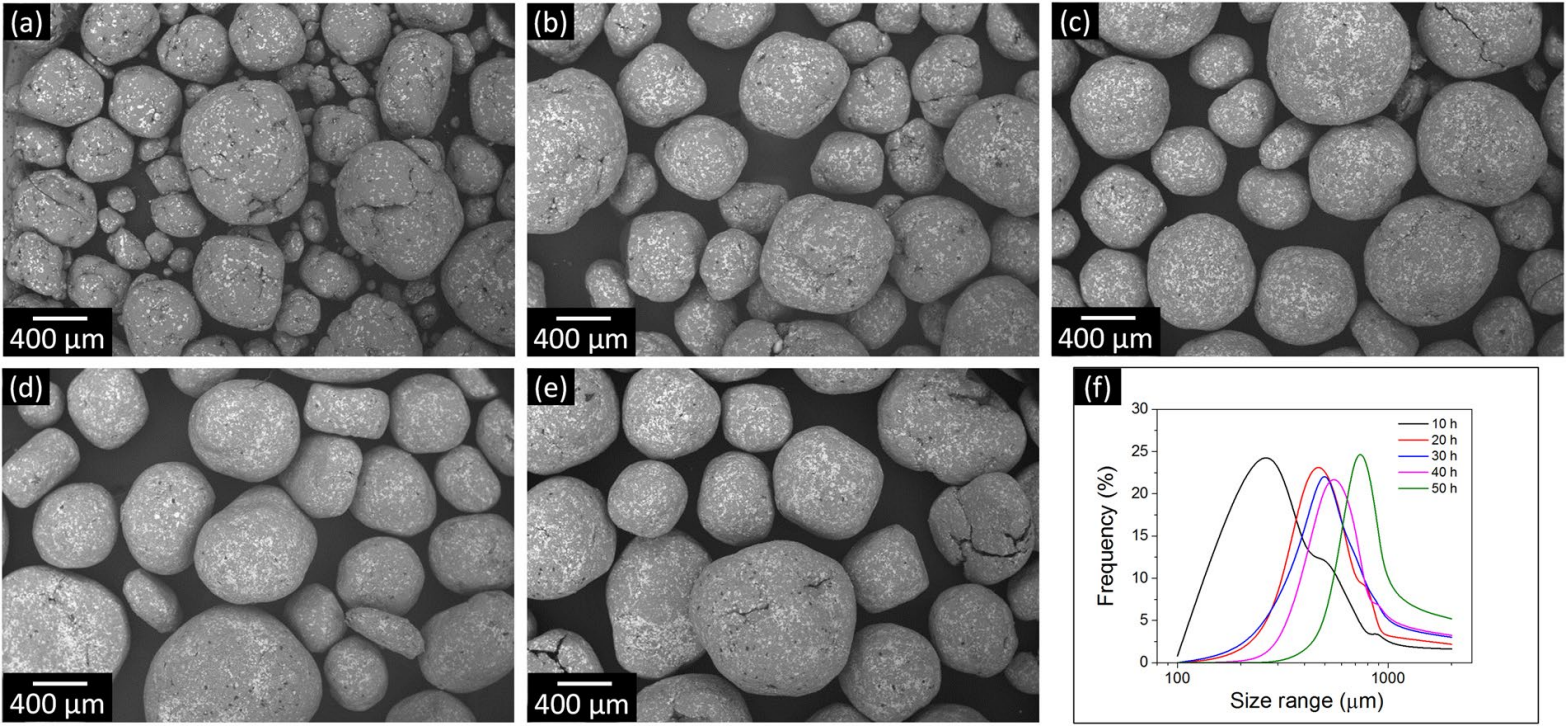
Effect of ball milling on particle size. SEM micrographs of the Al-Fe_3_Al powder mixtures milled for (**a**) 10, (**b**) 20, (**c**) 30, (**d**) 40 and (**e**) 50 h. (**f**) Particle size distributions of the powders milled for different periods.

In order to generate Al-Fe_3_Al composites with harmonic structures, powder mixtures consisting of pure aluminum [D(50) = 3 µm, Fig. 1a] and 20 vol.% of Fe_3_Al particles [D(50) = 10 µm, Fig. 1b] were ball milled for different periods (*t*_m_ = 1, 5, 10, 20, 30, 40 and 50 h). The SEM image of the Al-Fe_3_Al powder mixture milled for 1 h [Fig. 1c] shows that only a limited amount of powder clusters, consisting of Fe_3_Al particles trapped by aluminum, are formed at this stage. The increase of milling time to 5 h leads to the formation of larger clusters with a maximum size of about 140 µm along with comparatively less agglomerated aluminum and Fe_3_Al particles [Fig. 1d]. Clusters consisting exclusively of Fe_3_Al particles are not observed. Figure 2a–e display the SEM micrographs for the powder mixtures milled for *t*_m_ = 10, 20, 30, 40 and 50 h. After 10 h, only Al-Fe_3_Al clusters are visible and non-agglomerated aluminum or Fe_3_Al particles are not present anymore. The average size of these macro-particles increases with increasing milling time, while the particle size distributions become progressively narrower (Fig. 2f and Table 1).

**Table 1:** Effect of milling time on particle size, shell thickness and transformation temperature for the Al-Fe_3_Al powder mixtures.

Milling time (h)	Particle size D(50) (μm)	Shell thickness (μm)	Transformation temperature (K)
1	—	—	769
10	300	8 ± 0.6	747
20	490	15 ± 1.3	730
30	510	21 ± 0.8	722
40	570	27 ± 1.5	712
50	800	33 ± 1.6	702

**Figure 3:**
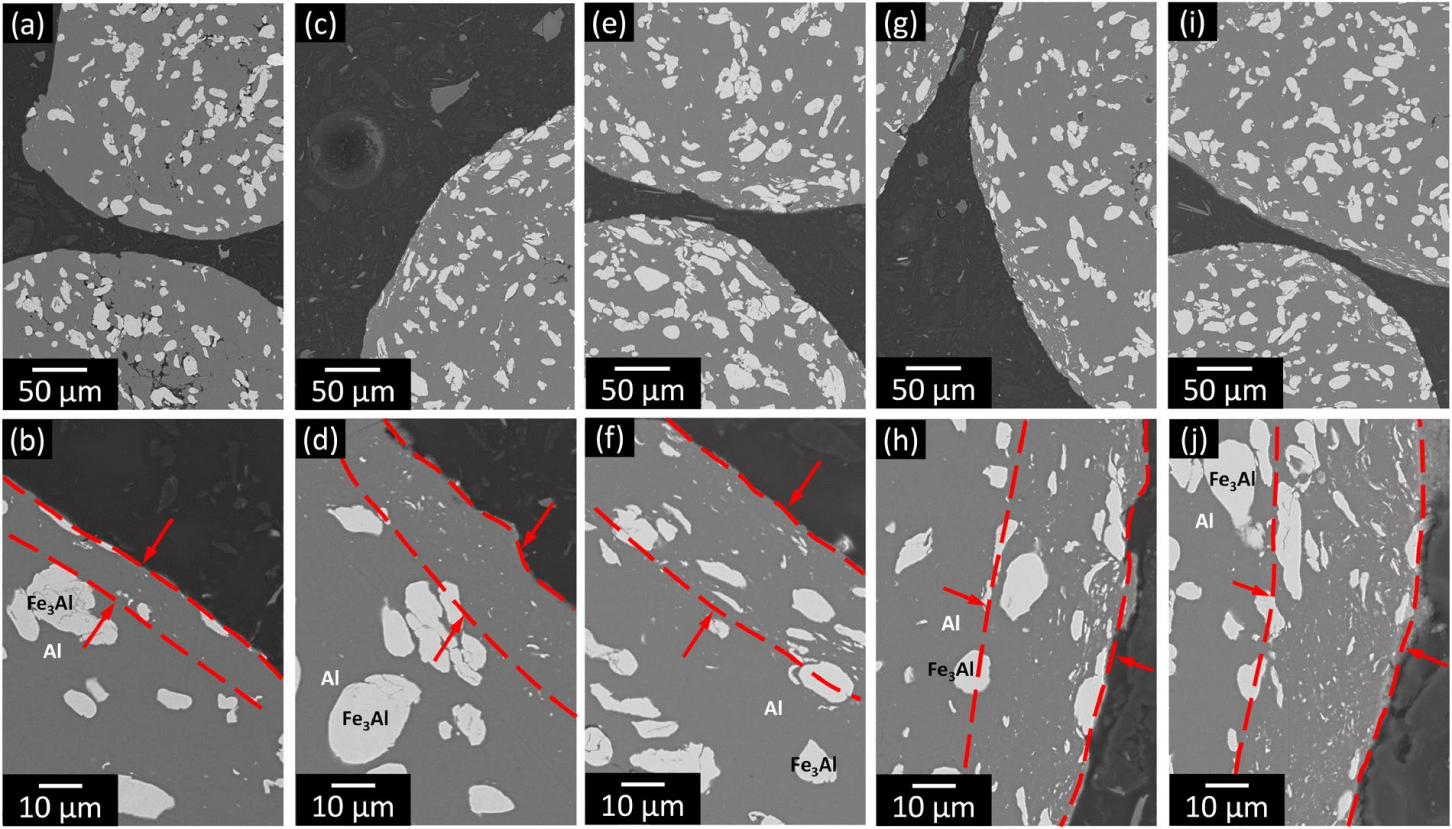
Effect of ball milling on particle microstructure. SEM images of the polished cross-section of the Al-Fe_3_Al powder mixtures milled for (**a**,**b**) 10, (**c**,**d**) 20, (**e**,**f**) 30, (**g**,**h**) 40 and (**i**,**j**) 50 h revealing the formation of a refined microstructure on the particle surface.

The characteristic microstructure of the macro-particles is reported in Fig. 3, which shows the SEM images of the polished cross-section of the milled powders as a function of the milling time for *t*_m_ ≥ 10. Every macro-particle displays a core consisting of relatively large Fe_3_Al particles embedded in the Al matrix, which is surrounded by a shell made of Al and small, mostly sub-micron, Fe_3_Al particles. In the core, the dispersion of Fe_3_Al is fairly homogeneous and the presence of submicron Fe_3_Al particles is rare. This indicates that after the formation of the macro-particles at *t*_m_ = 10 h the microstructural refining induced by ball milling is limited to their surface. Here, the Fe_3_Al particles are progressively fragmented by the action of the milling media and the thickness of the shell increases with increasing the milling time (Table 1). The preferential microstructural refinement limited to the surface of the particles is a necessary prerequisite for the generation of harmonic structures during subsequent powder consolidation^51^.

**Figure 4:**
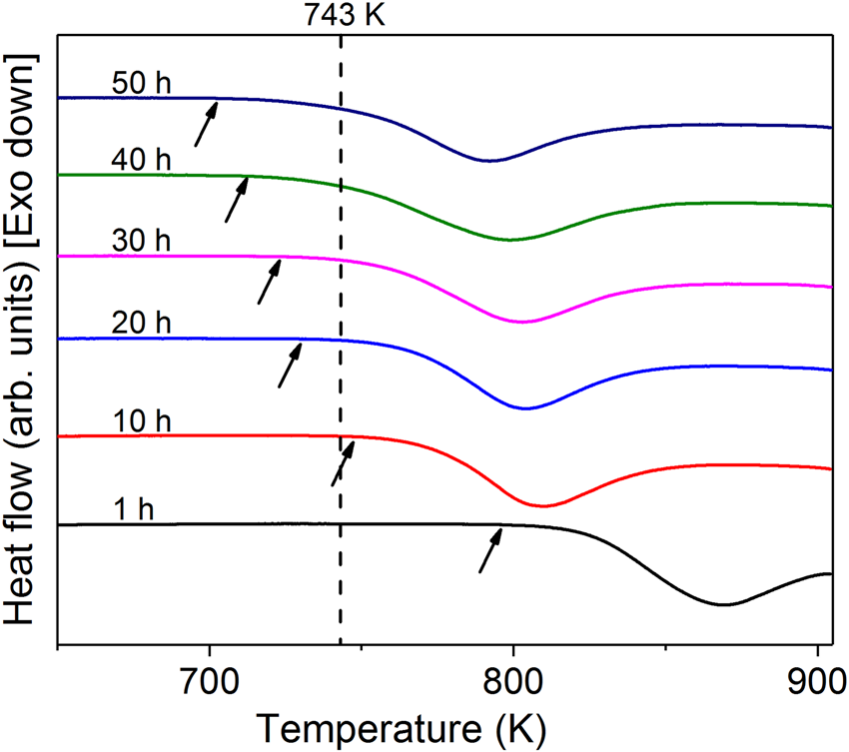
Thermal stability of the milled powders. DSC scans (heating rate 20 K/min) of the Al-Fe_3_Al powder mixtures milled for 1, 10, 20, 30, 40 and 50 h.

The combination of Al and Fe_3_Al phases in the present composites are not in a stable configuration and, when heated to high temperatures, they react to form Al_5_Fe_2_ and Al_13_Fe_4_ intermetallics^40^. The knowledge of the influence of milling on the thermal stability of the Al-Fe_3_Al mixtures is thus a prerequisite for properly controlling the subsequent consolidation step. To address this aspect, we performed DSC experiments on cold-pressed specimens obtained from powders mixtures milled from 1 to 50 h. Cold pressing ensures the formation of interfaces between the macro-particles, allowing atomic diffusion and, consequently, the phase transformation during heating, while preserving the as-milled microstructure. Figure 4 displays the DSC scans of the cold-pressed composites. In the temperature range investigated here, the DSC curves exhibit one exothermic peak, indicative of a phase transformation. It has been reported that during heating of aluminum-Fe_3_Al composites, aluminum diffuses into Fe_3_Al particles and creates Al_5_Fe_2_ as first intermetallic reaction product at low sintering temperatures^40^. Here, the formation of Al_5_Fe_2_ is shifted toward lower temperatures with increasing milling time, as shown by the change of the onset temperature of the transformation *T*_x_ (i.e. the temperature at which the heat flow signal diverge from the baseline^60^) marked by arrows in Fig. 4. The increase of milling time from 1 to 50 h decreases *T*_x_ by ~67 K (Table 1).

The decrease of the transformation temperature can be ascribed to the energy accumulated in the milled powder. During milling, powder particles are subjected to high-energy impacts when trapped between the milling media^61^. The energy transferred to the particles at the impacts leads to work-hardening and fracture. Additionally, milling generates a variety of crystal defects, including dislocations, vacancies and stacking faults as well as new surfaces and grain boundaries^61–63^. As a results, a significant amount of enthalpy, which can reach values of about 40% of the heat of fusion^64^, can be stored in the milled material. The stored enthalpy is then released during heating at high temperatures due to defect recovery and grain growth^65^, which can in turn remarkably decrease the characteristic temperature of a reaction^66–68^, as observed here.

**Figure 5:**
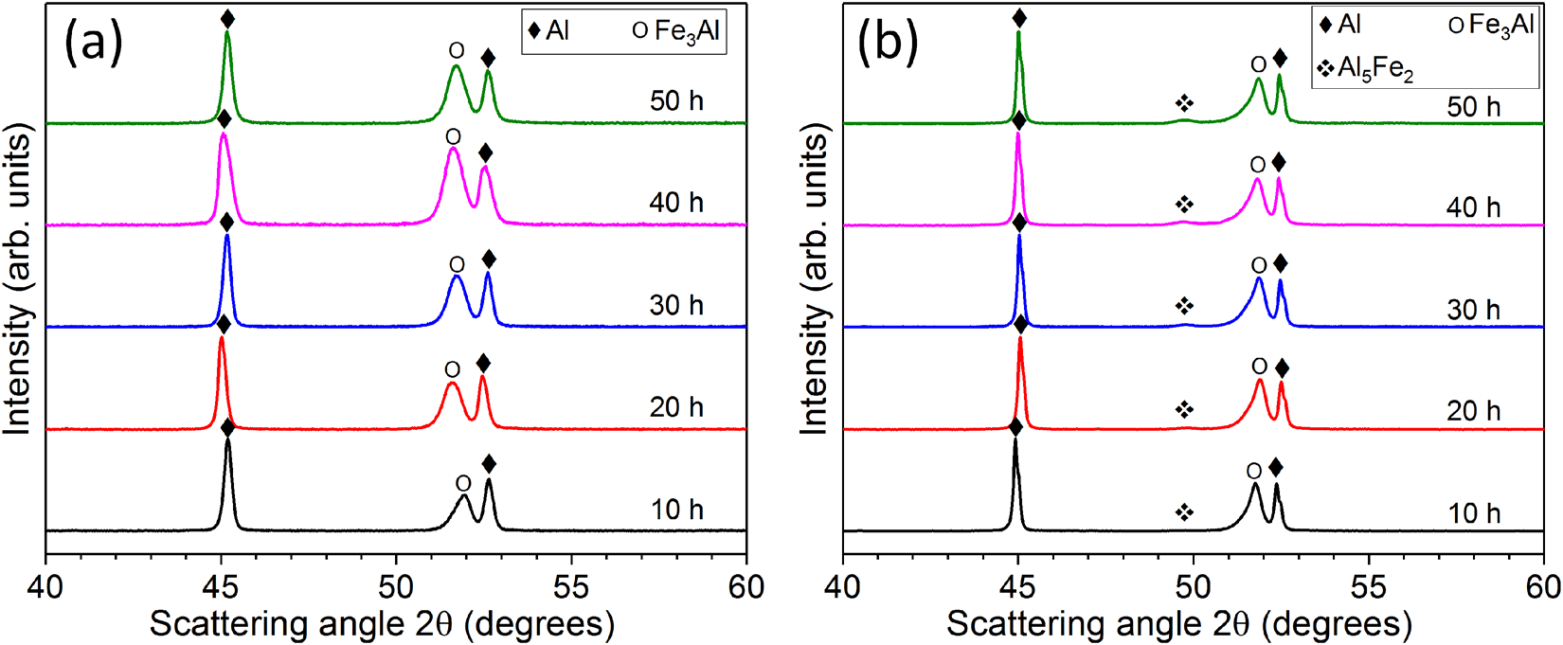
Effect of ball milling on phase formation in the particulate precursors and bulk composites. XRD patterns (λ = 0.179 nm) for (**a**) the ball-milled Al-Fe_3_Al powder mixtures and (**b**) bulk composite specimens synthesized by hot pressing of the milled powders.

**Figure 6:**
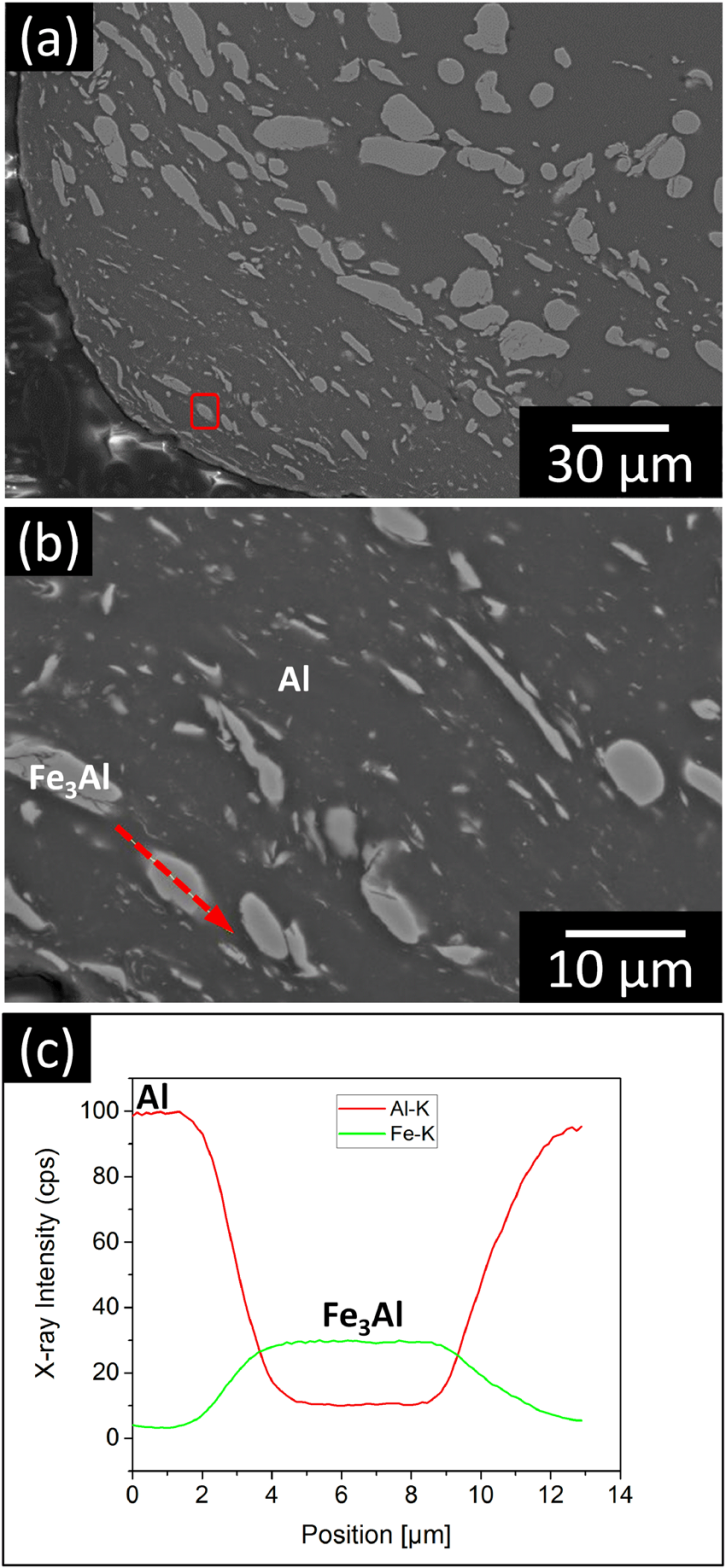
Compositional analysis of matrix-reinforcement interface. SEM images (**a**,**b**) and EDX elemental concentration profiles (**c**) for the Al-Fe_3_Al powder mixture milled for 50 h. The area shwon in (**b**) corresponds to the red box in (**a**). The dashed red arrow in (**b**) represents the scanned EDX line.

Figure 5a displays the XRD patterns for the powder mixtures milled for different times. The patterns exclusively show the presence of Al and D03-type Fe_3_Al phases, which indicates that no new phases are generated during milling. The patterns exhibit peak broadening with the increase of milling time from 10 to 50 h for both aluminum and Fe_3_Al peaks, which can be attributed to the reduction of the reinforcement and matrix particle size (especially in the shells of the composite macro-particles, Fig. 3) and to the increase in defect density and lattice strain^43,69–72^. The absence of a mechanically-induced reaction between Al matrix and Fe_3_Al reinforcement is corroborated by the SEM micrographs and EDX elemental concentration profiles of the powder milled for 50 h displayed in Fig. 6a–c: no additional phases, beside Al and Fe_3_Al, are detected, not even in the shell of the macro-particles which is presumably at a higher energy level due to the reduced size and presence of a high defect density.

## Bulk composites by hot-pressing of milled Al-Fe_3_Al powders

The XRD patterns for the hot-pressed AMCs [Fig. 5b] reveal the presence of aluminum and Fe_3_Al in all samples. In addition, the AMCs show that a small amount of orthorhombic Al_5_Fe_2_ phase is also formed. The peak intensity of the Al_5_Fe_2_ phase slightly increases with the increase of milling time, suggesting that an increasing amount of Al_5_Fe_2_ is formed. This behavior can be ascribed to the diminished temperature *T*_x_ (where the Al_5_Fe_2_ phase starts to form) displayed in Fig. 4, which becomes progressively lower than the hot pressing temperature (743 K).

**Figure 7:**
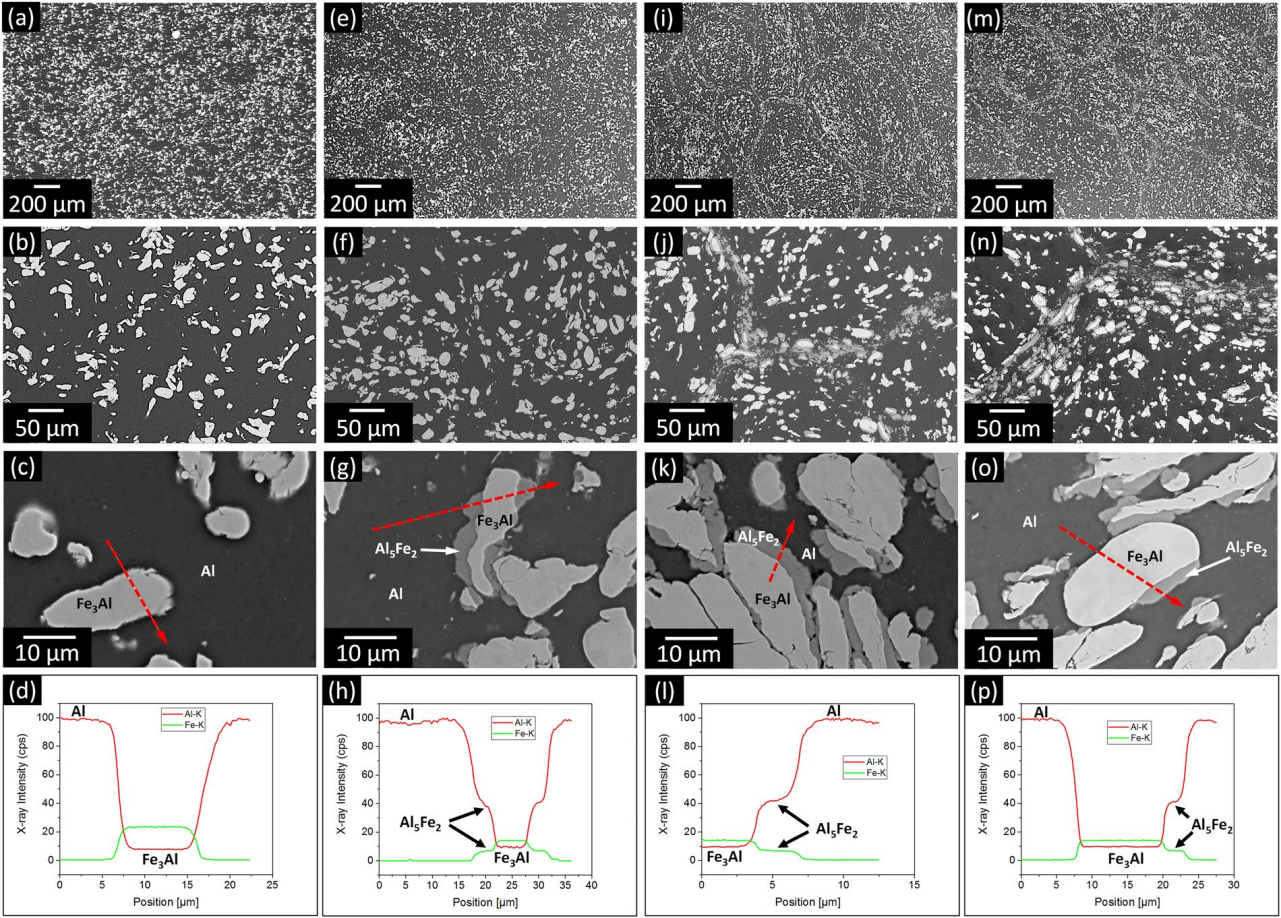
Microstructure of the bulk composites. SEM images and EDX elemental concentration profiles for the AMCs synthesized by hot pressing of the powder mixtures milled for (**a–d**) 1, (**e–h**) 10, (**i–l**) 40 and (**m–p**) 50 h. The dashed red arrows represent the scanned EDX lines.

Figure 7 shows the SEM images and EDX elemental concentration profiles for the AMCs produced by hot pressing of the composite powders milled for *t*_m_ = 1, 10, 40 and 50 h. The bulk specimens exhibit a harmonic-type microstructure with features resembling the parent milled macro-particles: macro-areas consisting of large and relatively undeformed Fe_3_Al particles embedded into the Al matrix that are surrounded by an interface where the size of the Fe_3_Al particles is reduced. EDX analysis indicates that Al_5_Fe_2_ is not formed in the AMC synthesized from the powder milled for 1 h. On the other hand, the AMCs consolidated from the powders milled for *t*_m_ ≥ 10 h show the formation of the Al_5_Fe_2_ phase at the interface between Fe_3_Al and aluminum matrix, confirming the results from XRD in Fig. 5b. The Al_5_Fe_2_ phase is typically formed at the interface between the parent macro-particles. This behavior can be attributed to the effect of milling, which is localized here to the surface of the particles (Fig. 3). As a result, the microstructure of the surface is considerably refined and supposedly has a higher defect density than the core of the macro-particles. During hot pressing, the higher enthalpy stored in the surface would, therefore, locally induce the formation of the Al_5_Fe_2_ phase at lower temperatures compared with the less defective cores, explaining the present results. As a result of the increased shell thickness in the parent particles (Fig. 3), the thickness of the refined interface in the composites increases with milling time and reaches a maximum of about 50 μm after milling for 50 h. In contrast to the other specimens, the refined interface between macro-areas is not continuous in the 50 h AMC and pores with size of 5–10 µm size are observed. This is reflected in the lower relative density of this sample (96.7 ± 0.4%) compared with the composites synthesized from the powders milled for *t*_m_ < 50 h (98.8 ± 0.5%).

**Figure 8:**
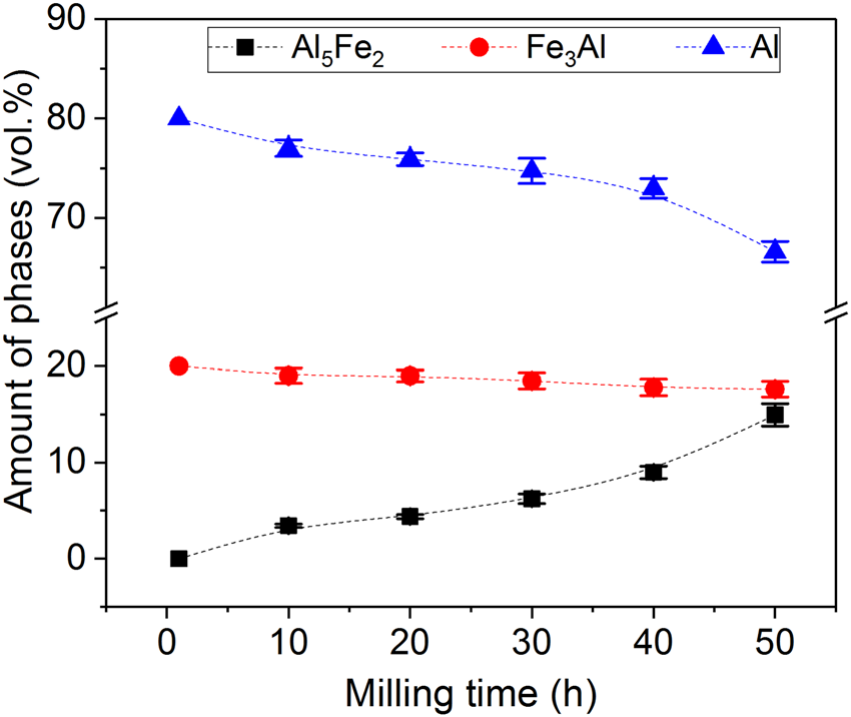
Effect of ball milling on amounts of phases in the bulk composites. Volume percent of the different phases characterizing the hot-pressed composites as function of the milling time showing the progressive formation of the Al_5_Fe_2_ phase.

The amount of phases characterizing the different composites evaluated by SEM is shown in Fig. 8. The volume fraction of Al_5_Fe_2_ increases with increasing milling time (along with the thickness of the shells of the parent macro-particles) and, accordingly, the volume fraction of aluminum and Fe_3_Al decreases. The volume fraction of Al_5_Fe_2_ is highest in the 50 h AMC (15 ± 1 vol.%); this may explain the large residual porosity in this specimen as a result of the consolidation temperature used, being too low to properly sinter FeAl-rich samples^73^.

## Mechanical properties of hot-pressed Al-Fe_3_Al composites

**Figure 9:**
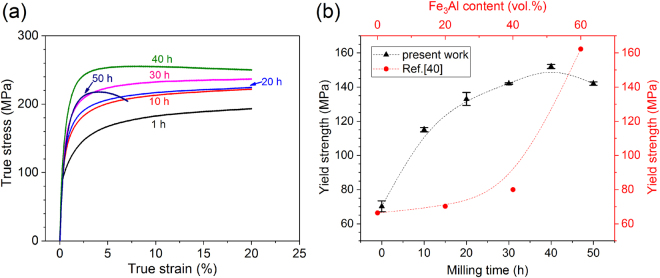
Effect of milling on mechanical properties. (**a**) Room temperature compressive stress-strain curves for the hot-pressed AMCs obtained from the powder milled for 1, 10, 20, 30, 40, and 50 h. (**b**) Yield strength of the present bulk Al-Fe_3_Al composites (initial Fe_3_Al content of 20 vol.%) as a function of the milling time and of the Al-Fe_3_Al composites reinforced with different Fe_3_Al contents (data from ref.^40^).

**Figure 10:**
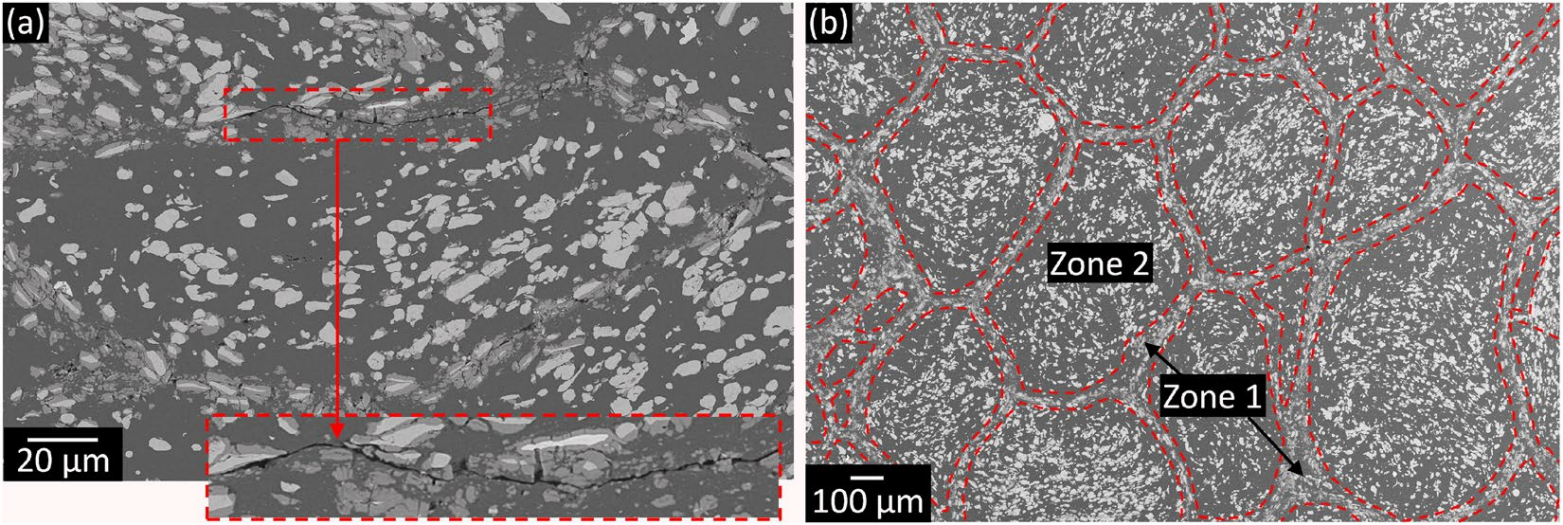
Microstructural features of the harmonic composites. (**a**) SEM images after compression test of the AMC synthesized from the powder milled for 50 h revealing preferential crack formation along the Al_5_Fe_2_-rich refined matrix. (**b**) Representative micrograph of the harmonic structures generated in the bulk Al-Fe_3_Al composites consisting of a continuous matrix with refined reinforcement (zone 1) that encloses macro-regions with coarser and rather homogeneously distributed reinforcing particles (zone 2).

The mechanical behavior of the AMCs consolidated from the powders milled for 1 to 50 h were investigated by using room temperature compression tests. The stress-strain curves are shown in Fig. 9 along with the corresponding yield strength (0.2% offset). The strengthening effect induced by milling is quite remarkable: the yield strength of the composites increases from 70 ± 3 MPa for the 1 h AMC to 152 ± 1 MPa for the 40 h AMC and then it decreases to 142 ± 1 MPa for the 50 h AMC, most likely because of the higher residual porosity of this sample. The strength of the present 40 h AMC is only 10 MPa lower than the composite reinforced with 60 vol.% of Fe_3_Al fabricated from powder mixtures milled for only 1 h^40^ (red points in Fig. 9b). Given that the total amount of reinforcement (Fe_3_Al + Al_5_Fe_2_) in the 40 h AMC is only 27 vol.% (Fig. 8), the observed strengthening can be ascribed to the microstructural changes induced by milling the powder mixtures. All composites show no less than 20% strain, where the compression test was stopped, except for the 50 h AMC, which displays fracture at about 7% strain. Again, the reduced plastic deformation can be attributed to the residual porosity along with the large volume fraction of brittle Al_5_Fe_2_ formed during hot pressing at the interfaces of the macro-particles. The brittleness of these areas is illustrated in Fig. 10a: cracks preferentially form and propagate through the Al_5_Fe_2_-rich boundaries between the parent macro-particles.

## Correlations between harmonic structure and mechanical behavior

**Figure 11:**
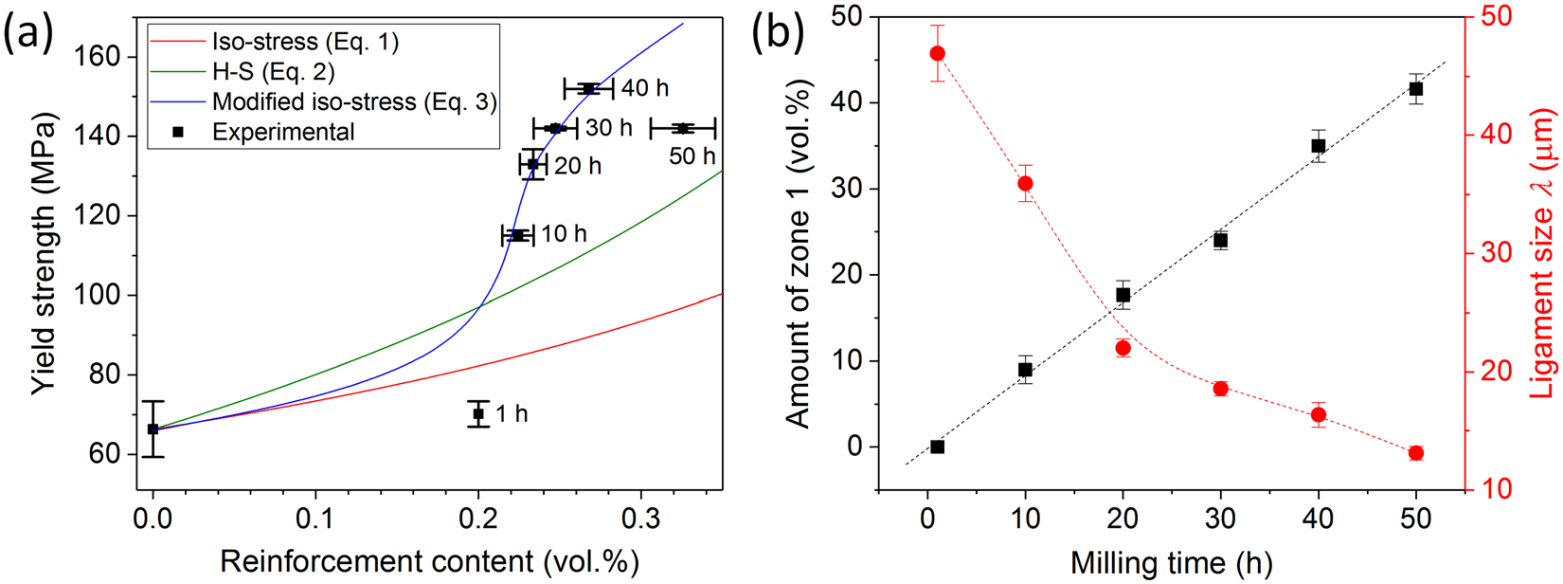
Modeling of the strength of the composites. (**a**) Yield strength of the composites as a function of the volume fraction of reinforcement (cumulative amount of Fe_3_Al and Al_5_Fe_2_): experimental data (points) and calculated values (lines) from the iso-stress model (equation 1), Hashin and Shtrikman (H-S) model (equation 2) and from the modified iso-stress model obtained by implementing the volume fraction of zone 1 (equation 3). (**b**) Amount of zone 1 and matrix ligament size of the harmonic composites as a function of the milling time.

The strength of composites reinforced with small volume fractions of reinforcement, where the distance between the reinforcing particles is large, can be well described by the lower bound of the rule of mixtures (RoM), the iso-stress model, which assumes that the matrix and reinforcement experience the same stress^10,74–76^ as:

1 $${\sigma }_{c}={(\frac{{V}_{r}}{{\sigma }_{r}}+\frac{{V}_{m}}{{\sigma }_{m}})}^{-1},$$

where *V* is the volume fraction, *σ* is the strength and the subscripts *c*, *r* and *m* indicate the composite, the reinforcement and the matrix, respectively. Indeed, the variation of the yield strength for the Al-Fe_3_Al composites with the Fe_3_Al particles uniformly distributed in the aluminum matrix is in good agreement with the trend predicted by the iso-stress model^40^. On the other hand, the strength of the present composites significantly diverges from the values predicted by the iso-stress model for *t*_m_ > 1 h: Equation 1 (red curve in Fig. 11a) progressively underestimates the experimental values of yield strength with increasing milling time. Similarly, the lower bound of the Hashin and Shtrikman (H-S) model:

2 $${\sigma }_{c}={(\frac{{\sigma }_{r}[{\sigma }_{r}{V}_{r}+{\sigma }_{m}(1+{V}_{m})]}{{\sigma }_{m}{V}_{r}+{\sigma }_{r}(1+{V}_{m})})}^{-1},$$

where the soft continuous matrix encapsulates the hard reinforcement^77^, does not properly describe the present results (green curve in Fig. 11a). This indicates that calculations solely based on the volume fraction of reinforcement are not capable to predict the strengthening effect in the present materials.

This behavior can be ascribed to the bimodal composite microstructure, which is harmonic-like and consists of a continuous matrix made of composite areas with refined reinforcement (zone 1 in Fig. 10b) that encloses macro-regions with coarser and rather homogeneously distributed reinforcing particles (zone 2 in Fig. 10b). Zone 1 is most likely stronger than zone 2 because of the high hardness of the Al_5_Fe_2_ phase^78^ and of the refined microstructure. The extent of zone 1 increases with increasing the milling time (Fig. 11b), which would explain the higher strength than predicted by Equations 1 and 2. The strengthening contribution of the areas comprising the refined reinforcement (zone 1) can be taken into account by modifying Equation 1 as:

3 $${\sigma }_{c}=(1+{V}_{z1}^{1/3}){(\frac{{V}_{r}}{{\sigma }_{r}}+\frac{{V}_{m}}{{\sigma }_{m}})}^{-1},$$

with *V*_z1_ being the volume fraction of zone 1 and where the choice of the cube root is in accordance with the model proposed by Gurland^79^ for describing the strength of WC-Co alloys.

The values of yield strength calculated using Equation 3 are in good agreement with the experimental data except for the material milled for 50 h (blue curve in Fig. 11a), where the residual porosity and crack formation decrease the strength of the material. For small values of *V*_z1_ (*t*_m_ ≤ 1 h), the strength follows the iso-stress model, as observed for Al-Fe_3_Al composites with *V*_z1_ = 0^40^; the strength of the composites then raises with increasing *V*_z1_ for *t*_m_ > 1 h. Equation 3 of course cannot be employed to describe the strength of composites with *V*_z1_ approaching unity; in this case, the strengthening contribution of zone 1 will be overestimated by a factor of 2.

In order to accurately describe the strength of the present composites, therefore, both the volume fraction of reinforcement and the characteristic microstructural features describing the harmonic structure (here the extent of zone 1) have to be taken into account. Two main aspects make the choice of the amount of zone 1 the most suitable descriptive feature of the present harmonic structures. The first aspect is related to the formation of a continuous refined matrix (i.e. zone 1), which behaves like a stiff skeleton that encapsulates the softer and coarser macro-regions (zone 2) and limits their deformation in response to the applied load, increasing the overall strength. The second aspect involves the strengthening contribution resulting from the reduction of the matrix ligament size (λ), which is indirectly considered by implementing *V*_z1_ in Equation 1. The amount of fragmented Fe_3_Al particles with reduced size increases with *V*_z1_, which consequently reduces λ in zone 1. At the same time, the size of zone 2 progressively decreases, giving rise to an overall reduction of λ in the entire specimen, as shown in Fig. 11b.

## Summary

The effectiveness of the creation of harmonic structures as a method to strengthen Al-based metal matrix composites has been evaluated. To achieve this aim, Al-Fe_3_Al composites with a bimodal microstructure consisting of a continuous fine-grained matrix that encloses macro-regions with coarser reinforcing particles have been synthesized by consolidation of the powder mixtures ball milled for different periods. The amount of the refined matrix, and consequently the strength of the harmonic composites, increases with increasing the milling time up to 50 h, when the higher residual porosity and the formation of cracks during mechanical loading decrease the strength of the material. For *t*_m_ < 50 h, the generation of the bimodal microstructure induces a significant strengthening of the harmonic composites: the strength exceeds that of the conventional material by a factor of 2, while retaining considerable plastic deformation. Modeling of the mechanical properties indicates that the strength of the present harmonic composites can be accurately described by taking into account both the volume fraction of reinforcement and the characteristic microstructural features describing the harmonic structure.

## Methods

Powder mixtures consisting of pure aluminum and 20 vol.% of Fe_3_Al particles were ball milled for different milling periods (*t*_m_ = 1, 5, 10, 20, 30, 40 and 50 h) under argon atmosphere using a Retch PM 400 planetary ball mill and hardened steel balls and vials operating at a rotation speed of 100 rpm. The ball diameter was 10 mm and the ball to powder weight ratio was 10:1. Milling was stopped every 15 minutes for an interval of 15 minutes to avoid any excessive temperature rise during the process. The milled powders were consolidated by hot pressing at room temperature and 743 K under argon atmosphere by applying 640 MPa uniaxial pressure for 10 minutes. The microstructure of the milled powders was investigated by scanning electron microscopy (SEM) using a Hitachi TM-1000 table top microscope. Ten to fifteen SEM micrographs were analyzed for every specimen using the software ‘ImagJ’ and the cumulative size distribution curves (cumulative frequency percent as a function of the particle size range^6^) were used to calculate the population based D(50) size of the milled powders. The thermal stability of the composites consolidated at room temperature was studied by differential scanning calorimetry (DSC) using a Netzsch DSC 404 C calorimeter (heating rate 20 K/min). Phase identification of the milled powders and hot-pressed AMCs was carried out by X-ray diffraction (XRD) using a D3290 PANalytical X’pert PRO diffractometer with Co-*Kα* radiation (λ = 0.179 nm). A scanning electron microscope GEMNI 1530 equipped with an energy dispersive X-ray spectrometer (EDX) was used to characterize the microstructure of the hot-pressed AMCs. Thirty to fifty SEM images for every composite were examined by using the image analyzer software ‘ImageJ’ in order to evaluate the amount of the different phases. The Archimedes method was used to measure the densities of the hot-pressed AMCs. The matrix ligament size (*λ* = *L*/*N*, where *N* is the number of matrix region intercepts on the test line of length *L*^80^) characterizing the composites was calculated from the arithmetic mean of seventy measurements by superimposing random lines on seven SEM micrographs. Room temperature quasistatic compression tests (strain rate = 8 × 10^−5^ s^−1^) were performed on cylindrical samples (6 mm length and 3 mm diameter) of the hot-pressed composites using an Instron 5869 testing facility. The strain was measured directly on the samples using a Fiedler laser-extensometer. The compression tests for all composites (except the 50 h milled AMC) were intentionally stopped at 20% strain. At least four specimens for each composite were tested in order to ensure the reproducibility of the results. The amount of oxygen, evaluated by carrier gas hot extraction using a Leco ON-836 analyzer, was found to be 0.495 ± 0.011 wt.% in the pure Al powder and 0.692 ± 0.005 wt.% in the Fe_3_Al particles, which gives a value of 0.570 ± 0.012 wt.% in the initial Al-Fe_3_Al powder mixture. The oxygen content slightly increases to 0.607 ± 0.006 wt.%, 0.627 ± 0.009 wt.% and 0.616 ± 0.020 wt.% in the powders milled for 20, 40 and 50 h, respectively. The strengthening contribution resulting from oxide formation can, therefore, be neglected.
